# Ambient UVR and Environmental Arsenic Exposure in Relation to Cutaneous Melanoma in Iowa

**DOI:** 10.3390/ijerph19031742

**Published:** 2022-02-03

**Authors:** Marvin E. Langston, Heidi E. Brown, Charles F. Lynch, Denise J. Roe, Leslie K. Dennis

**Affiliations:** 1 Division of Research, Kaiser Permanente Northern California, Oakland, CA 94612, USA; 2 Department of Epidemiology and Biostatistics, Mel and Enid Zuckerman College of Public Health, University of Arizona, Tucson, AZ 85724, USA; heidibrown@arizona.edu (H.E.B.); droe@arizona.edu (D.J.R.); ldennis@arizona.edu (L.K.D.); 3 Department of Epidemiology, College of Public Health, University of Iowa, Iowa City, IA 52242, USA; charles-lynch@uiowa.edu

**Keywords:** arsenic, melanoma, sun exposure, ultraviolet radiation

## Abstract

Intermittent sun exposure is the major environmental risk factor for cutaneous melanoma (CM). Cumulative sun exposure and other environmental agents, such as environmental arsenic exposure, have not shown consistent associations. Ambient ultraviolet radiation (UVR) was used to measure individual total sun exposure as this is thought to be less prone to misclassification and recall bias. Data were analyzed from 1096 CM cases and 1033 controls in the Iowa Study of Skin Cancer and Its Causes, a population-based, case-control study. Self-reported residential histories were linked to satellite-derived ambient UVR, spatially derived environmental soil arsenic concentration, and drinking water arsenic concentrations. In men and women, ambient UVR during childhood and adolescence was not associated with CM but was positively associated during adulthood. Lifetime ambient UVR was positively associated with CM in men (OR for highest vs. lowest quartile: 6.09, 95% confidence interval (CI) 2.21–16.8), but this association was not as strong among women (OR for highest vs. lowest quartile: 2.15, 95% CI 0.84–5.54). No association was detected for environmental soil or drinking water arsenic concentrations and CM. Our findings suggest that lifetime and adulthood sun exposures may be important risk factors for CM.

## 1. Introduction

The incidence of cutaneous melanoma (CM) rates in the US have increased from 9.75 per 100,000 in 1975 to 32.6 per 100,000 in 2017 among whites [1]. Intermittent sun exposure and sun sensitivity factors are the most important and well-described risk factors for CM [2,3,4]. CM risk, as with most cancers, increases with age, and incidence is higher among women than men before age 50. After age 50, incidence is higher in men [1]. Sex may represent differing patterns of exposure to ultraviolet radiation (UVR) [5,6] since melanomas appear most often on the head and neck or trunk of men and on the legs of women [7,8,9,10]. Melanomas on other regions, such as mucosal surfaces, are quite rare [11]. 

Sun sensitivity is the major host risk factor for skin cancers [12], and UVR is the major environmental risk factor implicated for skin cancers [2,13,14]. Intermittent sun exposure has been consistently shown to increase CM risk; however, chronic sun exposure has been inconsistently associated with CM [14,15,16,17]. Intermittent sun exposure typically includes sunburns and sunny vacations among sun sensitive individuals living in low UVR areas. Sun sensitivity measures are host factors associated with CM including skin color, hair color, eye color, tendency to sunburn, inability to tan, and skin type [12]. Other risk factors for CM include older age [1], family history of melanoma [12], and higher number of nevi [15].

Various other risk factors have been examined either with inconsistent results or by a limited number of studies. Occupational exposure studies of CM have looked at mostly indoor vs. outdoor occupations. A pooled analysis found occupational sun exposure to have a protective odds ratio [16], which may reflect self-selection to outdoor occupations by skin type. A cohort study of Iowa pesticide applicators (mostly farmers) and their spouses found obesity or body surface area at age 20, red hair, and use of sun protection in the sun during the growing season to increase the risk of CM, whereas spending > 10 h per day in the sun during the growing season was protective [18]. When restricted to pesticide applicators, associations were seen with benomyl and maneb/mancozeb fungicides and with carbaryl and parathion insecticides with a suggested association with lead arsenate insecticide and any arsenic pesticide [19]. A study in Hawaii found no association with CM and dietary lipids but did find a significant trend with increased alcohol intake [20].

Historically, epidemiological studies of CM have relied on using “time spent outdoors” and other distant recalled factors to measure sun exposure. Since recall of childhood, adolescent, and young adult exposures are difficult, with potential for differential bias between cases and controls, there is a need for more objective reporting of UVR exposure. Objective measures based on residential histories will eliminate most differential recall bias and should reduce non-differential misclassification of exposure as residential histories are easier to recall than lifestyle exposures. Residential histories have become important in cancer research [21,22]. Residential histories have been used for geospatial modeling to estimate arsenic water source histories [23], arsenic concentrations in private well water based on the nearest neighbor [24], and satellite imagery as a proxy for farmland [25]. They have also been used to examine space time clusters of cancer [26,27,28,29]. Several CM studies have shown an association with early migration (childhood) to sunny places [30,31,32,33], but findings among studies using lifetime measures of UVR have been inconsistent due to varying exposure assessment methods for sun exposure or UVR [20,34,35,36,37,38,39,40]. It is thought that inconsistencies may be due to host susceptibility in the form of sun sensitivity that may not have been accounted for, or may reflect differences in intermittent versus chronic sun exposure [41]. 

Remotely sensed ambient UVR data have displayed a high correlation to personal dosimetry [42,43]. Thus, use of such data could simplify survey data to residential histories, reduce the recall burden of study subjects, and strengthen the validity of exposure comparison across studies. US satellite-derived erythemal UVB from 1978–2014 were shown to have minimal changes over time [44], representing smaller changes than are necessary for appreciable misclassification of lifetime sun exposure based on the current UVR exposure literature, so they can be used to estimate ambient UVR. 

While UVR is the strongest etiologic agent of CM, other environmental agents, such as arsenic, have been associated with an increased risk of non-melanoma skin cancers [45,46,47,48]. However, few studies have examined arsenic in association with CM, and results thus far have been inconsistent [49,50,51]. Nevertheless, some researchers have hypothesized that arsenic may interact with UVR as evidenced by a stronger effect of UVR in populations exposed to high levels of arsenic [52,53]. Mechanistically, arsenic may serve as a co-carcinogen by inhibiting DNA damage repair processes through the enzyme PARP1, thereby exacerbating UVR-induced DNA damage [54,55]. 

The objective of this research was to examine the relationships between ambient UVR, environmental soil arsenic, and drinking water arsenic exposures on CM incidence. In this study, lifetime sun exposure is measured objectively using remotely sensed ambient UVR at places of residence in association with CM. Spatially derived arsenic concentrations in environmental soil and drinking water were included to provide a novel arsenic exposure measurement and examine the utility of this measure among a stable population.

## 2. Materials and Methods

### 2.1. Cases and Controls

Eligible cases were men and women newly diagnosed (recruited within 12 months of diagnosis) with microscopically confirmed CM from January 2010 through December 2012. All cases were semi-rapidly reported to the Iowa Cancer Registry. CM cases were contacted and interviewed by Iowa Cancer Registry staff, who were blinded to study hypotheses. A total of 1096 cases was analyzed in this study. Population-based controls were recruited using the Iowa Voter Registration List. Prior to beginning this study, the 2005 Iowa Voter Registration file was compared to the 2000 Iowa Census data. The Voter list contained 86.6% of the number of individuals listed in the census restricted to age 20 or older. Analogous to cases, all controls were age 20 years or older, residents of Iowa, and able to speak English. A total of 1033 unmatched controls were analyzed in this study. 

Individuals 20 years and over and living in Iowa at the time of recruitment were eligible for this population-based case-control study. Potential subjects were mailed a letter describing the study and thereafter contacted by phone. Subjects had to be available for a computer-assisted telephone interview in English. The computer-assisted telephone interview began with residential histories generating responses for city and state from birth to interview location, and date including month and year of each move. The survey then included questions regarding primary drinking water sources, decades of sun exposure, sunburn history, use of tanning beds, self-tanning cream use, sun sensitivity, family history of skin cancer, and basic demographic information. Approval for the study was obtained by the Institutional Review Boards of the University of Iowa and the University of Arizona. All participants provided informed consent. 

### 2.2. Residential Histories and Ambient UVR Assessment

Solar UVR data were obtained from NASA’s TOMS [56] and the Ozone Monitoring Instrument [57]. The TOMS system has been collecting ground-level UVR data aboard various satellites since 1978. From 2004 onward, the Ozone Monitoring Instrument has captured UVR estimates. Both systems provide continuous UVR data across the entire globe. UVR was estimated through noontime ultraviolet ground-level irradiance integrated throughout the day [58]. This model accounted for cloud conditions, ozone column, length of day, solar zenith angle, surface albedo (snow or forest cover), and atmospheric aerosols. Additionally, the UVR data used for this analysis were erythemic-weighted by the reference erythemal action spectrum, a measure of potential for UVR biological erythemal damage (skin redness) of fair skin [59].

Thirty years of monthly averages by location, spanning the time period 1978–2012 (368 months of total UVR data), were used to assign ambient UVR exposure to all subjects based on self-reported residential histories (city and state). Averaging over 30 years was justified due to small short-term variation in UVR [44]. To calculate the cumulative load of UVR, integrated daily averages were summed by month across the year. Individual ambient UVR estimates were assigned to study subjects for each year of life. These estimates were subsequently summed over specific life periods (childhood age 0–<14, adolescence age 14–18, adulthood >18, and lifetime) of each individual to achieve an overall erythemal UVR exposure assessment. Quartiles were set based on exposure in the control group prior to stratifying by any potential effect modifiers.

These analyses were conducted considering ambient sun exposure as potential sun exposure at place of residence to understand if locations matter and to avoid differential recall bias of recalled hours outdoors. Any bias on residential histories is expected to be minimal non-differential bias as total years at places of residence summed to the participants’ ages within one year.

### 2.3. Arsenic Measurements

Arsenic levels were examined by linking current residence at interview to arsenic sources from water and soil. Soil concentrations of arsenic were a magnitude higher than for water and are reported in parts per million instead of per billion. Participants were asked if their primary drinking water came from public supply, a private well, or bottled water. For individuals with private wells, we tested their well water for arsenic. For others, we used data collected by researchers at the University of Iowa Center for Health Effects of Environmental Contamination (CHEEC). 

Public drinking water: The United States Environmental Protection Agency (EPA) regulates arsenic concentration in public drinking water sources under the Safe Drinking Water Act, and as of January 2001, changed the maximum containment level (MCL) of arsenic from 0.05 parts per million (ppm) to 0.01 ppm [60,61]. Arsenic exceeding this level was considered high in the current study. Arsenic concentrations in public drinking water sources were assessed using the CHEEC database for study subjects who indicated using public sources for drinking water. This database contains 3167 unique historical arsenic results from September 1974 through December 2011. CHEEC uses these data to assess trends in water quality in Iowa and to verify that public water sources are meeting standards set by the EPA under the Safe Drinking Water Act. We calculated the average arsenic concentration from January 2001 through December 2011 from 656 unique locations throughout Iowa to estimate the cross-sectional arsenic concentration for public drinking water.

Private drinking water: Study subjects who indicated a private well as the current primary drinking water source during the computer-assisted telephone interview were mailed water sample packets. Returned samples of drinking water were sent to the University Hygienic Lab in Coralville, Iowa for arsenic testing. Private well samples (*n* = 318) were cross-sectional measurements with no repeated samples. Samples may represent long-term arsenic concentration in these systems as private wells are not regulated by the EPA’s 0.01 ppm arsenic regulation. Therefore, arsenic concentrations from private wells tested from 2010–2014 may be comparable to concentrations in public water sources from an earlier time period. 

For geospatial analysis, public water sources were either georeferenced to the centroid of the municipal area defined by the 2010 U.S. Census or georeferenced using the coordinates of the water source provided to the EPA’s facility registry system through linkage by the public water source’s Safe Drinking Water Act identification number. The private well samples were geo-referenced to the centroid of the study subject’s home zip code, ensuring privacy of subject-specific private well arsenic values, especially in sparsely populated areas. For all analyses, sampled sites with undetectable arsenic values were imputed for arsenic by triangular approximation, where samples were assigned the minimum detectable limit of the test divided by the square root of 2 [62]. Producers of bottled water (e.g., mineral water, purified water, spring water) must test regularly for arsenic and meet the EPA’s MCL [63,64]. Subjects who indicated bottled water as their primary drinking water source were assigned arsenic of minimum detectable limit divided by the square root of 2. For individual participants, their estimate came from the relevant data based on their statement of the current water source being public supply, private well or bottled water. Arsenic concentrations for public drinking water at the current location were assigned for study subjects who reported unknown primary sources of drinking water.

Environmental Soil: Currently, there is no nationwide EPA regulation regarding concentration of arsenic in soil across the US. Arsenic is regulated at the state level, and 45 states, including Iowa, have arsenic surface soil regulatory guidance values (RGV) [65]. The RGV for arsenic in Iowa (17 parts per million; ppm) is in the top third of least conservative state RGVs, well above the mean (11 ppm) and geometric mean (2.98 ppm) of state RGVs [65,66]. Soil arsenic concentrations were taken from the Iowa Statewide Trace Element Sampling Project conducted by the United States Geological Survey, May through August 2003 [67]. Field crews collected 532 sample sets across Iowa, including one from topsoil (0–20.3 cm deep) and one from subsoil (30.5–61 cm deep) at randomly selected geo-referenced locations throughout the state. The topsoil and subsoil samples were in the same location 100% of the time. Due to the spatial similarities between both sample types, only subsoil was considered in this analysis. Soil arsenic concentrations were determined by Atomic Absorption Spectroscopy methods [68]. Concentrations were 100% detectable with the detection limits of the test ranging from 0.6 ppm to 20 ppm. Arsenic concentrations in soil exceeding 11 ppm were considered high exposures in our study. 

Spatial interpolation between points was performed in ArcGIS 10.1 (ESRI, Redlands, CA, USA) for both the soil and water arsenic geo-referenced samples to create continuous spatial surfaces of arsenic concentration across Iowa. Interpolation was conducted via ordinary kriging, which is a method of estimation at unsampled locations using “nearest neighbors” weighted by a distance function [69]. The weights assigned were not based on distance alone, but also on minimizing the error variance of the entire model and spatial dependence within the sampled measures. The final variogram or function describing the extent of spatial dependence was chosen based on reducing the root-mean-square error of cross validation. Final models were chosen based on the average standard error closest to zero and the root mean square-standardized error closest to one [70]. Models, including anisotrophy (which allows spatial dependence to vary in different directions), were tested but did not result in improvement to the cross-validation results. 

### 2.4. Statistical Analysis

The bivariate associations between cumulative UVR at various life time periods and CM were assessed. Logistic regression was used to estimate the odds ratios (ORs) between arsenic in water and environmental soil near the current residence, and CM. Due to the low risk of CM in African Americans and Asians [1,71], and small percentages in the Iowa population (3% and 2%, respectively), they were excluded from these analyses. Continuous exposures were grouped based on quartiles of exposure in controls. To understand the joint effects and the impact of regulation of environmental arsenic exposure from contaminated soil and drinking water on CM, subjects were categorized into four categories based on the MCL in drinking water and mean RGV in the US for soil. A linear test of trend was also estimated for all models using ordinal data. The current residence was used for the primary arsenic analysis. In a sensitivity analysis, to estimate the effects of a more chronic arsenic exposure assessment, we restricted the analysis to subjects residing more than 5 years in their current location. 

Effect modification of ambient UVR and CM by sex, sun sensitivity factors, and arsenic levels was examined. For arsenic and CM, effect modification by sex, body mass index (BMI), and education level was explored. Potential confounding effects of age, sex, sun sensitivity factors, education, BMI, sunny vacations, and tanning bed use were examined [72]. Confounding was determined based on a change in the OR of interest (ambient UVR, soil arsenic or water arsenic) of 15%. Two-tailed *p*-values < 0.05 were considered significant. All statistical analyses were conducted in SAS 9.3 (SAS Institute, Cary, NC, USA). This study was approved by the Institutional Review Board at the University of Arizona (200711703).

## 3. Results

CM cases were older, slightly more likely to be male, and less likely to be a college graduate (Table 1). Study participants were relatively stable with 87%, 74%, and 64% living in the current residential location for at least 5 years, 10 years, and 15 years, respectively. Marital status was not associated with CM. Those who always burn, but never tan had a nearly three-fold increased OR compared to those who rarely burn and tan easily. A two-fold greater OR was seen for those who reported some sort of burning then tanning. Other sun sensitivity factors, including skin color, eye color, and hair color had increased associations with CM for those who were sun sensitive (lighter complexions compared to dark). As expected, cases were more likely to have a family history of skin cancer (OR = 1.36; 95% CI: 1.13–1.64).

### 3.1. Ambient UVR and Cutaneous Melanoma

We observed a significant interaction effect for only ambient UVR and sex (*p* < 0.001) in the analyses completed, therefore ambient UVR analyses are presented stratified by sex. Among men, ambient UVR during childhood (ages less than 14) and during high school were not associated with CM (Table 2). An increased OR was seen with UVR after age 18. CM was associated with lifetime ambient UVR with an OR of 6.89 for UVR > 37,868 kJ/m^2^ compared to UVR < 25,659 kJ/m^2^ (95% CI: 2.33–20.3; Table 2). Similar to men, no associations with UVR during childhood (ages less than 14) or high school were seen for women. CM was associated with UVR after age 18 in women (OR 2.94 95% CI: 1.38–6.27). However, lifetime ambient UVR showed non-significant increases in CM for women (Table 2). Moving to Iowa during the 5 years before interview from a location with less or more average ambient UVR exposure than Iowa was not associated with CM in either women or men. 

### 3.2. Environmental Arsenic Exposure and Cutaneous Melanoma

The primary drinking water source type (public vs. bottled or private well) at the current residence was not associated with CM. Table 3 presents risk of CM for arsenic in soil and water by quartiles of exposure in the controls and exceeding the regulatory guidance in arsenic content. No associations were seen. 

To estimate the effects of a more chronic arsenic exposure, we restricted the analysis to those residing more than 5 years in their current location, and the results were largely unchanged. However, when water filtration was restricted to use of a reverse osmosis filter, this appeared to be protective of CM with an OR of 0.65 (95% CI: 0.46–0.94) among subjects who lived in their current residence more than five years. No association was seen for combinations of water and soil estimates that exceeded the MCL of 0.01 ppm or RGV of 11 ppm, respectively. No effect modification by arsenic concentration in soil or water was observed for lifetime ambient UVR and CM in either men or women (Table 4).

## 4. Discussion

Lifetime ambient UVR exposure and exposure age 18+ were associated with CM in this study, whereas childhood and adolescent exposures were not. Among women, adulthood ambient UVR exposure was associated with CM, but childhood, and adolescent exposures were not and lifetime was only suggestive. This may reflect low cumulative ambient UVR in a population like Iowa, that may take decades of exposure to accumulate enough UVR to increase risk for CM. Other studies of ambient UVR and CM similarly did not find a consistent association for childhood and adolescent exposures [73,74]. Tatalovich et al. [75] found a slightly positive association between adolescent and early adulthood ambient UVR and CM in a Los Angeles-based case-control study, while a positive association for ambient UVR at birth and at 10 years of age for multiple primary melanomas was found among study subjects in the US, Australia, and Canada [76]. 

The almost 6-fold increase in OR among men with the highest quartile of UVR exposure provides evidence that lifetime ambient sun exposure may also be important for CM. For women, a slight association was suggestive. Iowa has a relatively moderate annual UVR. Using remotely sensed satellite data linked with the residential history of study subjects by month and year might reflect less exposure measurement error than self-reported measures, allowing for better estimation of risk. These results may also reflect fewer issues with recall bias. The large magnitude of effect found here was surprising considering the relatively stable study population of Iowa residents. When we restricted analyses to those who solely lived in Iowa throughout their life course, the magnitude of effect attenuated in men and increased in women. This suggests that the composition of the population moving into or out of Iowa may vary according to sex. Other studies have objectively measured lifetime UVR but found no significant associations for CM [73,74,77,78,79]. Cust et al. [73] found no association for lifetime UVR and melanomas diagnosed before age 40, while Fears et al. [77] found no association with lifetime cumulative UVR in men or women using a Philadelphia and San Francisco population. No association for CM was found for lifetime ambient UVR exposure among women in either the Nurses’ Health Study or the Women’s Health Initiative cohorts [79]. Although prospective, exposure may be misclassified in these studies as residential histories were not complete. Another study using TOMS satellite data for residents of western Washington found a 2-fold increase in risk of CM among women and no association among men [78]. This study displayed a large variability in UVR exposure, showing that movement of subjects from high UVR areas to western Washington explained some of the increase in CM in this lower UVR area. This may suggest that more women moved to Seattle from higher UVR areas. A study of nevi (precursors to melanoma) in Washington state conducted 16 years earlier also found the highest levels of nevi were among those who moved to the area from California [80]. In our study, men were more likely to move to Iowa from other places, and those who moved to Iowa were more likely to have high lifetime ambient UVR exposure than women who moved to Iowa. Women in the reference group migrated to Iowa from areas with lower exposure. This may explain the stronger association of ambient UVR in men. 

Our varied findings by sex may represent differing patterns of UVR exposure and disease between men and women. When age is considered, the incidence of CM in women is higher than men in young adulthood [81,82,83]. This may be due in part to a higher likelihood of indoor tanning among females, which is associated with increased CM risk [84,85,86]. Alternatively, after age 40–50, incidence rates reverse, with higher rates attributed to men [81,82,83]. Some have suggested that these changes in rates may be in part androgen driven [86,87,88], linked to increased time in the sun for occupational and recreational exposures, and decreased personal protective behaviors [89]. Other hypotheses include the structural and biological differences of skin, including the potential heightened capacity of DNA damage repair in females [90,91]. Findings using simulated UVR data revealed that the doses required to elicit a similar immunosuppression response following UVR exposure in men were 3 times lower than those in women [92].

The summary odds ratio for CM and total sun exposure from a meta-analysis of over 50 studies indicates no linear association [14]. This meta-analysis included studies of self-reported total sun exposure that may be prone to recall bias and measurement error biasing the results towards the null value. The magnitude of self-reported total or lifetime sun exposure and CM may be much closer to the null value than our results for ambient lifetime UVR for a few reasons. The use of ambient UVR gives increased power to detect an association and may reduce non-differential reporting of sun exposure. Measurement of total sun exposure over one’s life may take both accurate accounting of time spent outdoors in the sun, and enough within study population variation to determine an association if present. Those attempting to measure total sun exposure and CM should consider study population carefully and interpret findings more narrowly on a local scale. 

Uncertainty still exists for the role of ambient UVR on sun exposure measurement. While ambient UVR may be the potential for sun exposure, this measure captures aspects of incidental sun exposure. Self-reported measures of sun exposure based on recall, such as time spent outdoors, may only capture perceived, planned, or intentional exposures. Time spent outdoors may underestimate sun exposure by omitting incidental exposure, whereas ambient UVR may overestimate sun exposure since individuals in the same geographic location may have different sun seeking behaviors. This would explain the large magnitudes of effect seen here compared to studies reporting recalled sun exposure.

CM was not associated with spatially derived arsenic in drinking water or environmental soil at the residence of the subject’s interview. The lack of association seen here for arsenic and CM may be real or partially attributed to low endemic concentrations of arsenic in soil and water and/or misclassification of the exposure assessment. While exposure to arsenic has often been positively associated with non-melanoma skin cancers [45], the association with melanoma has been inconsistent [49,50,51]. In areas of low endemic arsenic concentrations, like much of the US, the hypothesized cancer risks may be from chronic exposures to low doses. Previous non-ecological studies using other surrogates of exposure, such as drinking water arsenic concentration [51] or municipal vs private water sources, have shown no association [93], while studies using toenails as a biomarker of total chronic exposure have produced mixed results [50,51]. Uncovering an association (if one exists) may depend on the methodological ability to estimate the true chronic arsenic dose. 

Due to lack of sufficient long-term arsenic data for all sources, we assumed arsenic concentrations in current drinking water represented chronic arsenic exposures. We were unable to explore this assumption for these data. Arsenic concentrations seen here may be lower than long-term estimates of arsenic in these drinking water sources, leading to misclassification of the arsenic exposure estimate. However, in analyses restricted to those residing more than 5 years in their current location, the results for arsenic were largely unchanged. This provides some support for more chronic exposure. The National Research Council suggested that arsenic exposures in drinking water required to cause cancer may be as short as 5 years [94]. We were unable to capture secondary water source information or drinking water sources outside the home. Therefore, our exposure assessment for water may not be complete. Exposure assessment in low endemic arsenic areas must be constructed carefully to avoid non-differential measurement error biasing estimates towards the null value. Here, recent arsenic measurements were assumed to represent historical exposure. That may be valid in soil, but arsenic in drinking water is more closely regulated in larger cities. Treatment and filtering of drinking water may also influence measurements over time. Here, we assumed that subjects in the same community would be drawing water from nearby public sources. If this assumption does not hold, then our exposure assessment for those using public water sources may be incomplete. Also, we were able to match the city name with 75% of study subjects who specified their drinking water source for the current residence, but the remaining 25% could have caused non-differential misclassification. 

We did find reduced odds ratio of CM in subjects that used a reverse osmosis filter. While reverse osmosis filters may reduce arsenic concentration in water by 75–99% [95,96,97], the protective effect may reflect a reduction in other water contaminants as reverse osmosis filters eliminate other trace elements and water contaminants that may be associated with CM, such as copper, which in one study was reported to be associated with melanoma [98]. As expected, those that lived in their current homes more than five years were more likely to use reverse osmosis filters (9% vs. 3%).

This study had several strengths, including population-based incident cancer cases and population-based controls. It also used an objective measure of sun exposure that has been shown to be a better predictor of sun exposure than sun sensitivity factors [12]. Using remotely sensed satellite UVR estimates in the future may allow for cheaper, more accurate, and more efficient analyses of sun exposure-related outcomes. Previous studies have suggested that those with lower lifetime ambient UVR exposure may be more likely exposed to higher levels of intermittent UV [73,74]. We were able to adjust for potential measures of intermittent sun exposure, including lifetime sunny vacations and lifetime tanning bed use. Other studies have examined lifetime ambient UVR and melanoma, but this is the first epidemiological study to look at possible effects modified by arsenic exposure. We did not find a significant interaction effect; however, this relationship may be better assessed in a high arsenic exposure area with a large gradient of exposures.

This study had a few limitations. Although residential history is matched with ambient UVR, individual behavior based on sun exposure knowledge or sun sensitivity factors may not reflect the ambient UVR estimates, which may lead to inaccurate of true sun exposure. While we were unable to examine the effects of occupational exposures (pesticides or chemicals) on the UVR and CM relationship, a study of Iowa pesticide applicators found no interaction between hours of sun exposure and pesticides (two fungicides and two insecticides) associated with CM [19]. For arsenic, missing private well arsenic concentrations were imputed using a spatially derived surface of arsenic in Iowa based on those subjects that supplied private well water for testing. These concentrations were geo-located to the zip code of the study subject’s current home. The accuracy of arsenic measurement may be impacted in areas where large intra zip code variation in arsenic is seen. Arsenic concentrations for public drinking water were assigned for study subjects who reported unknown primary sources of drinking water. Exclusion of these individuals in a sensitivity analysis did not appreciably change point estimates for arsenic analyses. We were not able to determine actual intake of water and consequently arsenic ingestion. We attempted to account for this by using filter-weighted estimates of arsenic exposure. Future studies should better measure intake of water along with the effect of water filtration and better measures of arsenic.

## 5. Conclusions

Our findings suggest that CM is associated with an increased odds ratio for adulthood ambient (age 18+) sun exposure in both women and men along with lifetime ambient sun exposure in men. This is important as most other studies of CM have only shown associations with intermittent exposure, such as sunburns or sunny vacations. Thus, potential lifetime sun exposure in sunny locations is important. Arsenic concentration in drinking water and environmental soil at place of residence were not associated with melanoma, but future studies should focus on better sampling and measurement of arsenic. Spatially derived estimates for ambient sun exposure should be used to examine risk for CM in future studies.

## Figures and Tables

**Table 1:** Demographic and sun sensitivity characteristics of 1096 cases and 1033 controls in the Iowa Study of Skin Cancer and Its Causes.

Host Factors	Cases		Controls		OR ^b^ (95% CI)
	*N*	% ^a^	*N*	% ^a^	
Age					
20–39	145	13	173	17	ref
40–49	147	14	156	15	1.13 (0.82–1.55)
50–59	227	21	258	25	1.03 (0.78–1.37)
60–69	256	23	265	26	1.11 (0.84–1.47)
70–79	199	18	136	13	1.69 (1.24–2.31)
80+	122	11	45	4	3.09 (2.05–4.65)
Trend OR ^c^					2.22 (1.65–2.98)
Sex					
Women	531	48	567	55	ref
Men	565	52	466	45	1.22 (1.03–1.46)
Marital Status					
Not married	269	25	263	26	ref
Married	822	75	766	74	1.08 (0.88–1.33)
Education					
Less than college	310	28	226	22	ref
Some college	312	29	288	28	0.91 (0.71–1.15)
College Graduate	469	43	514	50	0.79 (0.63–0.99)
Trend OR ^c^					0.78 (0.63–0.98)
Skin Type					
Rarely burn, tan easily	283	26	402	39	ref
Sometimes burn, then tan	483	44	406	39	2.07 (1.67–2.56)
Usually burn, tan with difficulty	223	21	162	16	2.35 (1.81–3.07)
Always burn, never tan	102	9	58	6	2.89 (2.00–4.18)
Trend OR ^c^					3.17 (2.33–4.30)
Skin Color					
Medium to Dark	238	22	332	32	ref
Fair	855	78	700	68	1.72 (1.41–2.10)
Eye color					
Black or Brown	379	35	450	44	ref
Green	165	15	150	15	1.38 (1.06–1.80)
Blue or Gray	549	50	427	41	1.50 (1.24–1.81)
Hair color					
Black or Brown	596	55	709	69	ref
Blond	287	27	223	22	1.68 (1.37–2.08)
Red	210	19	100	9	2.62 (2.00–3.42)
Family History of Skin Cancer					
No	605	57	625	62	ref
Yes	450	43	385	38	1.36 (1.13–1.64)

CI = confidence interval; *n* = number; OR= odds ratio; ref = reference. ^a^ Missing or unknown data are excluded from percentage calculations. ^b^ Adjusted for age and sex. ^c^ Trend OR fitting a line to β coefficients by category assuming an equal increase in the ln(OR) for each level comparing the first category to the last.

**Table 2:** Ambient erythemal UVR exposure by life period and risk of cutaneous melanoma, Iowa ^a^.

Ambient UVR (kJ/m^2^) (In Quartiles Defined by Controls)	Men			Women		
	Cases (*N* = 565)	Controls (*N* = 466)	OR ^b^ (95% CI)	Cases (*N* = 531)	Controls (*N* = 567)	OR ^b^ (95% CI)
<14 years old						
<11,280	125	127	ref	153	132	ref
11,280–12,878 ^c^	119	113	0.83 (0.57–1.25)	116	145	0.63 (0.43–0.92)
12,888–14,386	144	95	1.09 (0.72–1.65)	134	162	0.72 (0.48–1.06)
>14,386	177	131	0.75 (0.50–1.12)	128	128	0.82 (0.54–1.25)
Trend OR ^d^			0.82 (0.55–1.20)			0.86 (0.57–1.29)
14–18 years old						
<3293	125	115	ref	147	144	ref
3293–4266 ^c^	127	105	1.00 (0.67–1.47)	141	152	0.82 (0.58–1.16)
4267–4944	141	121	0.81 (0.55–1.20)	119	138	0.81 (0.55–1.18)
>4944	172	125	0.74 (0.50–1.11)	124	133	0.98 (0.66–1.46)
Trend OR ^d^			0.72 (0.49–1.06)			0.96 (0.66–1.41)
>18 years old						
<9276	67	109	ref	146	150	ref
9276–14,782	82	106	1.42 (0.68–2.97)	128	152	1.54 (0.89–2.70)
14,783–19,805 ^c^	153	129	2.42 (1.04–5.61)	109	128	2.12 (1.08–4.15)
>19,805	263	122	2.71 (1.09–6.74)	148	137	3.03 (1.39–6.62)
Trend OR ^d^			2.62 (1.17–5.85)			2.94 (1.38–6.27)
Lifetime						
<25,659	63	108	ref	147	151	ref
25,659–32,337 ^c^	93	115	2.27 (1.03–5.01)	133	143	1.48 (0.83–2.68)
32,338–37,868	146	131	4.43 (1.70–11.5)	107	127	1.83 (0.83–4.06)
>37,868	263	112	6.89 (2.33–20.3)	144	146	2.19 (0.85–5.65)
Trend OR ^d^			6.09 (2.21–16.8)			2.15 (0.84–5.54)

CI = confidence interval; kj = kilojoule; m^2^ = meters squared; *N* = number; OR= odds ratio; ref = reference; UVR = ultraviolet radiation. ^a^ Cumulative UVR by time period based on the months lived at each location and 30-year average erythemal UVR by month. ^b^ Adjusted for age, skin type, lifetime tanning bed use (women only), and lifetime sunny vacations. ^c^ Indicates average UVR exposure category for residents who have only ever lived in Iowa. ^d^ Trend OR fitting a line to β coefficients by category assuming an equal increase in the ln(OR) for each level comparing the first category to the last.

**Table 3:** Environmental soil arsenic combined spatially derived arsenic estimates, and risk of cutaneous melanoma, Iowa.

Environmental Exposures	Overall			>5 Years Current Location		
	Cases *N* = 1096	Controls *N* = 1033	OR ^a^ (95% CI)	Cases (*N* = 944)	Controls (*N* = 898)	OR ^a^ (95% CI)
Environmental soil As conc. (ppm)						
<7.82	312	257	ref	264	216	ref
7.82–9.00	247	252	0.82 (0.64–1.05)	212	222	0.80 (0.61–1.04)
9.01–9.73	283	264	0.92 (0.73–1.17)	244	225	0.93 (0.72–1.21)
>9.73	254	260	0.79 (0.62–1.01)	224	235	0.77 (0.59–1.00)
Trend OR ^b^			0.83 (0.66–1.05)			0.83 (0.65–1.06)
Soil As content exceeds RGV ^c^						
No	966	899	ref	832	773	ref
Yes	130	134	0.86 (0.66–1.11)	112	125	0.79 (0.60–1.05)
Drinking water As conc. (ppb)						
<3.04	273	257	ref	230	231	ref
3.04–3.54	242	259	0.90 (0.70–1.16)	211	217	0.99 (0.76–1.30)
3.55–4.74	309	248	1.18 (0.93–1.51)	265	212	1.25 (0.97–1.63)
>4.74	272	269	0.95 (0.74–1.21)	238	238	0.99 (0.77–1.29)
Trend OR ^b^			1.04 (0.82–1.30)			1.07 (0.83–1.37)
Drinking water As content exceeds MCL ^d^						
No	996	944	ref	852	819	ref
Yes	100	89	0.97 (0.71–1.32)	92	79	1.01 (0.74–1.40)
Used reverse osmosis filter						
No	1011	970	ref	864	844	ref
Yes	85	63	0.74 (0.52–1.04)	80	54	0.65 (0.46–0.94)
As content near current residence						
As_Water_ ≤ MCL/As_Soil_ ≤ RGV	891	829	ref	765	712	ref
As_Water_ ≤ MCL/As_Soil_ > RGV	105	115	0.79 (0.60–1.06)	87	107	0.71 (0.52–0.96)
As_Water_ > MCL/As_Soil_ ≤ RGV	75	70	0.88 (0.62–1.25)	67	61	0.89 (0.61–1.29)
As_Water_ > MCL/As_Soil_ > RGV	25	19	1.18 (0.64–2.17)	25	18	1.26 (0.68–2.36)
Trend OR ^b^			0.87 (0.60–1.27)			0.87 (0.58–1.29)

As = arsenic; CI = confidence interval; MCL = maximum contaminant level = 0.01 ppm; *N* = number; OR = odds ratio; ppm = parts per million; ref = reference; RGV, regulatory guidance value. ^a^ Age and sex adjusted. ^b^ Trend OR fitting a line to β coefficients by category assuming an equal increase in the ln(OR) for each level comparing the first category to the last. ^c^ Exceeding the mean US regulatory guidance value for arsenic in soil (11 ppm). ^d^ Exceeding the maximum containment level for arsenic in drinking water (0.01 ppm).

**Table 4:** Ambient lifetime UVR modified by spatially derived arsenic exposure and cutaneous melanoma, Iowa.

Ambient Lifetime UVR (kJ/m^2^) ^a^		Stratified by Soil As Concentration			
Stratified by Sex		Cases	Exceeds RGV ^b^ OR ^c^ (95% CI)	Cases	RGV Compliant OR ^c^ (95% CI)
Men:	<28,524	8	ref	84	ref
	28,525–35,600 ^d^	18	1.80 (0.43–7.47)	130	2.49 (0.98–6.31)
	>35,601	49	4.97 (1.16–21.3)	276	3.40 (1.41–11.3)
		*p*-value for interaction		*p* = 0.49	
Women:	<28,524	20	ref	180	ref
	28,525–35,600 ^d^	14	1.36 (0.44–4.27)	133	1.35 (0.64–2.87)
	>35,601	21	1.71 (0.50–5.77)	163	1.70 (0.69–4.22)
		*p*-value for interaction		*p* = 0.98	
		Stratified by Water As Concentration			
			Exceeds MCL ^e^		MCL Compliant
Men:	<28,524	7	ref	85	ref
	28,525–35,600 ^d^	13	1.31 (0.31–5.55)	135	1.73 (0.72–4.17)
	>35,601	38	2.95 (0.67–12.92)	287	2.71 (1.02–7.18)
		*p*-value for interaction		*p* = 0.77	
Women:	<28,524	19	ref	181	ref
	28,525–35,600 ^d^	10	0.86 (0.25–2.94)	137	1.28 (0.65–2.40)
	>35,601	13	0.55 (0.16–1.92)	171	1.58 (1.24–3.91)
		*p*-value for interaction		*p* = 0.13	

As = arsenic; CI = confidence interval; MCL = maximum contaminant level = 0.01 ppm; OR = odds ratio; ppm = parts per million; ref = reference; RGV, regulatory guidance value; UVR = ultraviolet radiation. ^a^ Cumulative UVR by time period based on the months lived at each location and 30-year average erythemal UVR by month, in tertiles defined by controls. ^b^ Exceeding the mean US regulatory guidance value for arsenic in soil (11 ppm). ^c^ Adjusted for age, skin type, lifetime tanning bed use, and lifetime sunny vacations. ^d^ Indicates average UVR exposure category for residents who have only ever lived in Iowa. ^e^ Exceeding the maximum containment level for arsenic in drinking water (0.01 ppm).

## Data Availability

Data for this study is available upon request to the senior author.

## References

[1] Howlader N., Noone A.M., Krapcho M., Miller D., Brest A., Yu M., Ruhl J., Tatalovich Z., Mariotto A., Lewis D.R. (2019). SEER Cancer Statistics Review, 1975–2017.

[2] Armstrong B.K., Kricker A. (2001). The epidemiology of UV induced skin cancer. J. Photochem. Photobiol. B Biol..

[3] El Ghissassi F., Baan R., Straif K., Grosse Y., Secretan B., Bouvard V., Benbrahim-Tallaa L., Guha N., Freeman C., Galichet L. (2009). A review of human carcinogens—Part d: Radiation. Lancet Oncol..

[4] Olsen C.M., Carroll H.J., Whiteman D.C. (2010). Estimating the attributable fraction for melanoma: A meta-analysis of pigmentary characteristics and freckling. Int. J. Cancer.

[5] Chang Y., Barrett J.H., Bishop D.T., Armstrong B.K., Bataille V., Bergman W., Berwick M., Bracci P.M., Elwood J.M., Ernstoff M.S. (2009). Sun Exposure and Melanoma Risk at Different Latitudes: A Pooled Analysis of 5700 Cases and 7216 Controls. Int. J. Epidemiol..

[6] Rastrelli M., Tropea S., Rossi C.R., Alaibac M. (2014). Melanoma: Epidemiology, Risk Factors, Pathogenesis, Diagnosis and Classification. In Vivo.

[7] Volkovova K., Bilanicova D., Bartonova A., Letašiová S., Dusinska M. (2012). Associations between environmental factors and incidence of cutaneous melanoma. Review. Environ. Health.

[8] Chiarugi A., Quaglino P., Crocetti E., Nardini P., De Giorgi V., Borgognoni L., Brandani P., Gerlini G., Manganoni A.M., Bernengo M.G. (2015). Melanoma density and relationship with the distribution of melanocytic naevi in an Italian population: A GIPMe study—The Italian multidisciplinary group on melanoma. Melanoma Res..

[9] Cho E., Rosner B.A., Colditz G. (2005). Risk Factors for Melanoma by Body Site. Cancer Epidemiol. Biomark. Prev..

[10] Yuan T.A., Lu Y., Edwards K., Jakowatz J., Meyskens F.L., Liu-Smith F. (2019). Race-, Age-, and Anatomic Site-Specific Gender Differences in Cutaneous Melanoma Suggest Differential Mechanisms of Early- and Late-Onset Melanoma. Int. J. Environ. Res. Public Health.

[11] Tyrrell H., Payne M. (2018). Combatting mucosal melanoma: Recent advances and future perspectives. Melanoma Manag..

[12] Gandini S., Sera F., Cattaruzza M.S., Pasquini P., Zanetti R., Masini C., Boyle P., Melchi C.F. (2005). Meta-analysis of risk factors for cutaneous melanoma: III. Family history, actinic damage and phenotypic factors. Eur. J. Cancer.

[13] MacKie R.M. (2006). Long-term health risk to the skin of ultraviolet radiation. Prog. Biophys. Mol. Biol..

[14] Gandini S., Sera F., Cattaruzza M.S., Pasquini P., Picconi O., Boyle P., Melchi C.F. (2005). Meta-analysis of risk factors for cutaneous melanoma: II. Sun exposure. Eur. J. Cancer.

[15] Gandini S., Sera F., Cattaruzza M.S., Pasquini P., Abeni D., Boyle P., Melchi C.F. (2005). Meta-analysis of risk factors for cutaneous melanoma: I. Common and atypical naevi. Eur. J. Cancer.

[16] Elwood J.M., Jopson J. (1997). Melanoma and sun exposure: An overview of published studies. Int. J. Cancer.

[17] Nelemans P., Rampen F., Ruiter D., Verbeek A. (1995). An addition to the controversy on sunlight exposure and melanoma risk: A meta-analytical approach. J. Clin. Epidemiol..

[18] Dennis L.K., Lowe J.B., Lynch C.F., Alavanja M.C. (2008). Cutaneous melanoma and obesity in the Agricultural Health Study. Ann. Epidemiol..

[19] Dennis L.K., Lynch C.F., Sandler D.P., Alavanja M.C. (2010). Pesticide use and cutaneous melanoma in pesticide applicators in the Agricultural Heath Study. Environ. Health Perspect..

[20] Le Marchand L., Saltzman B.S., Hankin J.H., Wilkens L.R., Franke A.A., Morris S.J., Kolonel L.N. (2006). Sun exposure, diet, and melanoma in Hawaii Caucasians. Am. J. Epidemiol..

[21] Jacquez G.M., Slotnick M.J., Meliker J.R., AvRuskin G., Copeland G., Nriagu J. (2011). Accuracy of Commercially Available Residential Histories for Epidemiologic Studies. Am. Epidemiol..

[22] Kanaroglou P., Delmelle E. (2016). Spatial Analysis and Health Geography.

[23] Meliker J.R., Slotnick M.J., AvRuskin G.A., Schottenfeld D., Jacquez G.M., Wilson M.L., Goovaerts P., Franzblau A., Nriagu J.O. (2010). Lifetime exposure to arsenic in drinking water and bladder cancer: A population-based case-control study in Michigan, USA. Cancer Causes Control.

[24] Meliker J.R., AvRuskin G.A., Slotnick M.J., Goovaerts P., Schottenfeld D., Jacquez G.M., Nriagu J.O. (2008). Validity of spatial models of arsenic concentrations in private well water. Environ. Res..

[25] Avruskin G.A., Meliker J.R., Jacquez G.M. (2008). Using satellite derived land cover information for a multi-temporal study of self-reported recall of proximity to farmland. J. Expo. Sci. Environ. Epidemiol..

[26] Nordsborg R.B., Meliker J.R., ErsbÃll A.K., Jacquez G.M., Poulsen A.H., Raaschou-Nielsen O. (2014). Space-time clusters of breast cancer using residential histories: A Danish case-control study. BMC Cancer.

[27] Nordsborg R.B., Meliker J.R., ErsbÃll A.K., Jacquez G.M., Raaschou-Nielsen O. (2013). Space-time clustering of non-Hodgkin lymphoma using residential histories in a Danish case-control study. PLoS ONE.

[28] Jacquez G.M., Barlow J., Rommel R., Kaufmann A., Rienti MJr AvRuskin G., Rasul J. (2013). Residential mobility and breast cancer in Marin County, California, USA. Int. J. Environ. Res. Public Health.

[29] Sloan C.D., Nordsborg R.B., Jacquez G.M., Raaschou-Nielsen O., Meliker J.R. (2015). Space-time analysis of testicular cancer clusters using residential histories: A case-control study in Denmark. PLoS ONE.

[30] Holman C.D., Armstrong B.K., Heenan P.J., Blackwell J.B., Cumming F.J., English D.R., Holland S., Kelsall G.R., Matz L.R., Rouse I.L. (1986). The causes of malignant melanoma: Results from the West Australian Lions Melanoma Research Project. Recent Results Cancer Res..

[31] Autier P., Doré J.F., Gefeller O., Cesarini J.P., Lejeune F., Koelmel K.F., Lienard D., Kleeberg U.R. (1997). Melanoma risk and residence in sunny areas. EORTC Melanoma Co-operative Group. European Organization for Research and Treatment of Cancer. Br. J. Cancer.

[32] Robsahm T.E., Tretli S. (2001). Cutaneous malignant melanoma in Norway: Variation by region of residence before and after the age 17. Cancer Causes Control.

[33] Mack T.M., Floderus B. (1991). Malignant melanoma risk by nativity, place of residence at diagnosis, and age at migration. Cancer Causes Control.

[34] Garbe C., Kruger S., Stadler R., Guggenmoos-Holzmann I., Orfanos C.E. (1989). Markers and relative risk in a German population for developing malignant melanoma. Int. J. Dermatol..

[35] Green A., Bain C., McLennan R., Siskind V. (1986). Risk factors for cutaneous melanoma in Queensland. Recent Results Cancer Res..

[36] Landi M.T., Baccarelli A., Tarone R.E., Pesatori A., Tucker M.A., Hedayati M., Grossman L. (2002). DNA repair, dysplastic nevi, and sunlight sensitivity in the development of cutaneous malignant melanoma. J. Natl. Cancer Inst..

[37] Rodenas J.M., Delgado-Rodriguez M.T., Herranz Tercedor J., Serrano S. (1996). Sun exposure, pigmentary traits, and risk of cutaneous malignant melanoma: A case-control study in a Mediterranean population. Cancer Causes Control.

[38] Loria D., Matos E. (2001). Risk factors for cutaneous melanoma: A case-control study in Argentina. Int. J. Dermatol..

[39] Zanetti R., Franceschi S., Rosso S., Colonna S., Bidoli E. (1992). Cutaneous melanoma and sunburns in childhood in a southern European population. Eur. J. Cancer.

[40] Zanetti R., Rosso S., Martinez C., Nieto A., Miranda A., Mercier M., Loria D.I., Østerlind A., Greinert R., Navarro C. (2006). Comparison of risk patterns in carcinoma and melanoma of the skin in men: A multi-centre case-case-control study. Br. J. Cancer.

[41] Armstrong B.K., Cust A.E. (2017). Sun exposure and skin cancer, and the puzzle of cutaneous melanoma: A perspective on Fears et al. Mathematical models of age and ultraviolet effects on the incidence of skin cancer among whites in the United States. American Journal of Epidemiology 1977; 105: 420–427. Cancer Epidemiol..

[42] Cahoon E.K., Wheeler D.C., Kimlin M.G., Kwok R.K., Alexander B.H., Little M.P., Linet M.S., Freedman D.M. (2013). Individual, environmental, and meteorological predictors of daily personal ultraviolet radiation exposure measurements in a united states cohort study. PLoS ONE.

[43] Sun J., Lucas R., Harrison S., Van Der Mei I., Armstrong B.K., Nowak M., Brodie A., Kimlin M.G. (2014). The relationship between ambient ultraviolet radiation (UVR) and objectively measured personal UVR exposure dose is modified by season and latitude. Photochem. Photobiol. Sci..

[44] Langston M.E., Dennis L.K., Lynch C.F., Roe D., Brown H.E. (2017). Temporal Trends in Satellite-Derived Erythemal UVB and Implications for Ambient Sun Exposure Assessment. Int. J. Environ. Res. Public Health.

[45] Surdu S., Fitzgerald E.F., Bloom M.S., Boscoe F.P., Carpenter D.O., Haase R.F., Gurzau E., Rudnai P., Koppova K., Févotte J. (2013). Occupational exposure to arsenic and risk of nonmelanoma skin cancer in a multinational European study. Int. J. Cancer.

[46] Gilbert-Diamond D., Li Z., Perry A.E., Spencer S.K., Gandolfi A.J., Karagas M.R. (2013). A population-based case-control study of urinary arsenic species and squamous cell carcinoma in New Hampshire, USA. Environ. Health Perspect..

[47] Leonardi G., Vahter M., Clemens F., Goessler W., Gurzau E., Hemminki K., Hough R., Koppova K., Kumar R., Rudnai P. (2012). Inorganic arsenic and basal cell carcinoma in areas of Hungary, Romania, and Slovakia: A case-control study. Environ. Health Perspect..

[48] Matthews N.H., Fitch K., Li W.Q., Morris J.S., Christiani D.C., Qureshi A.A., Cho E. (2019). Exposure to Trace Elements and Risk of Skin Cancer: A Systematic Review of Epidemiologic Studies. Cancer Epidemiol. Prev. Biomark..

[49] Baastrup R., Sørensen M., Balstrøm T., Frederiksen K., Larsen C.L., Tjønneland A., Overvad K., Raaschou-Nielsen O. (2008). Arsenic in drinking-water and risk for cancer in Denmark. Environ. Health Perspect..

[50] Beane Freeman L.E., Dennis L.K., Lynch C.F., Thorne P.S., Just C.L. (2004). Toenail arsenic content and cutaneous melanoma in Iowa. Am. J. Epidemiol..

[51] Yager J.W., Erdei E., Myers O., Siegel M., Berwick M. (2016). Arsenic and ultraviolet radiation exposure: Melanoma in a New Mexico non-Hispanic white population. Environ. Geochem. Health.

[52] Chen Y., Graziano J.H., Parvez F., Hussain I., Momotaj H., van Geen A., Howe G.R., Ahsan H. (2006). Modification of risk of arsenic-induced skin lesions by sunlight exposure, smoking, and occupational exposures in Bangladesh. Epidemiology.

[53] Melkonian S., Argos M., Pierce B.L., Chen Y., Islam T., Ahmed A., Syed E.H., Parvez F., Graziano J., Rathouz P.J. (2011). A prospective study of the synergistic effects of arsenic exposure and smoking, sun exposure, fertilizer use, and pesticide use on risk of premalignant skin lesions in Bangladeshi men. Am. J. Epidemiol..

[54] Cooper K.L., Yager J.W., Hudson L.G. (2014). Melanocytes and keratinocytes have distinct and shared responses to ultraviolet radiation and arsenic. Toxicol. Lett..

[55] Zhou X., Speer R.M., Volk L., Hudson L.G., Liu K.J. (2021). Arsenic co-carcinogenesis: Inhibition of DNA repair and interaction with zinc finger proteins. Semin. Cancer Biol..

[56] International Research Institute (2015). Nasa GSFC Total Ozone Mapping Spectrometer. http://iridl.ldeo.columbia.edu/SOURCES/.NASA/.GSFC/.TOMS/.

[57] Hovila J., Arola A., Tamminen J. (2013). OMI/Aura Surface UVB Irradiance and Erythemal Dose Daily L3 Global Gridded 1.0 Degree × 1.0 Degree V3, NASA Goddard Space Flight Center, Goddard Earth Sciences Data and Information Services Center (GES DISC). https://disc.gsfc.nasa.gov/datasets/OMUVBd_003/summary.

[58] Herman J., Celarier E. (2016). Erythemal Exposure Data Product. http://ozoneaq.gsfc.nasa.gov/media/docs/erynotes.pdf.

[59] McKinlay A., Diffey B. (1987). A reference action spectrum for ultraviolet induced erythema in human skin. CIE J..

[60] US EPA, US Environmental Protection Agency (2001). Technical Fact Sheet: Final Rule for Arsenic in Drinking Water. http://nepis.epa.gov/Exe/ZyPdf.cgi?Dockey=20001XXE.txt.

[61] US EPA, US Environmental Protection Agency (2003). Arsenic Treatment Technology Evaluation Handbook EPA 816-r-03-014. https://frtr.gov/pdf/arsenicdesignmanualpeerreviewdraft.pdf.

[62] Hornung R.W., Reed L.D. (1990). Estimation of average concentration in the presence of nondetectable values. Appl. Occup. Environ. Hyg..

[63] IBWA, International Bottled Water Association (2020). Bottled Water Code of Practice. https://bottledwater.org/wp-content/uploads/2020/12/IBWA-MODEL-CODE-2020-Rev-2020-FINAL.pdf.

[64] US FDA, US Food and Drug Administration (2015). Code of Federal Regulations Title 21 Sec. 165.110 Bottled Water. https://www.accessdata.fda.gov/scripts/cdrh/cfdocs/cfcfr/CFRSearch.cfm?fr=165.110.

[65] Jennings A.A. (2010). Analysis of regulatory guidance values for residential surface soil arsenic exposure. J. Environ. Eng..

[66] IDNR (2021). Statewide Standards for Contaminants in Soil and Groundwater. Iowa Department of Natural Resources. https://programs.iowadnr.gov/riskcalc/home/statewidestandards.

[67] Rowden R., The Iowa State-Wide Trace Element Soil Sampling Project: Design and Implementation (2010). Iowa Department of Natural Resources. https://www.iihr.uiowa.edu/igs/publications/uploads/2014-08-24_08-08-51_ofr-2010-1.pdf.

[68] Haswell S.J. (1991). Atomic Absorption Spectrometry: Theory, Design and Applications.

[69] Oliver M.A., Webster R. (1990). Kriging: A method of interpolation for geographical information systems. Int. J. Geogr. Inf. Syst..

[70] Johnston K., Ver Hoef J.M., Krivoruchko K., Lucas N. (2001). Using ArcGIS Geostatistical Analyst. http://dusk.geo.orst.edu/gis/geostat_analyst.pdf.

[71] Census, US Census Bureau (2016). Annual Estimates of the Resident Population by Sex, Race, and Hispanic Origin for the United States, States, and Counties: 1 April 2010 to 1 July 2015. https://www.census.gov/programs-surveys/popest.html.

[72] Berwick M., Buller D.B., Cust A., Gallagher R., Lee T.K., Meyskens F., Pandey S., Thomas N.E., Veierød M.B., Ward S., Kaufman H., Mehnert J. (2016). Melanoma Epidemiology and Prevention. Melanoma. Cancer Treatment and Research.

[73] Cust A.E., Jenkins M.A., Goumas C., Armstrong B.K., Schmid H., Aitken J.F., Giles G., Kefford R., Hopper J.L., Mann G. (2011). Early-life sun exposure and risk of melanoma before age 40 years. Cancer Causes Control.

[74] Ransohoff K.J., Ally M.S., Stefanick M.L., Keiser E., Spaunhurst K., Kapphahn K., Pagoto S., Messina C., Hedlin H., Manson J.E. (2016). Impact of residential UV exposure in childhood versus adulthood on skin cancer risk in Caucasian, postmenopausal women in the women’s health initiative. Cancer Causes Control.

[75] Tatalovich Z., Wilson J.P., Mack T., Yan Y., Cockburn M. (2006). The objective assessment of lifetime cumulative ultraviolet exposure for determining melanoma risk. J. Photochem. Photobiol. B Biol..

[76] Kricker A., Armstrong B.K., Goumas C., Litchfield M., Begg C.B., Hummer A.J., Marrett L.D., Theis B., Millikan R.C., Thomas N. (2007). Ambient UV, personal sun exposure and risk of multiple primary melanomas. Cancer Causes Control.

[77] Fears T.R., Bird C.C., Guerry D., Sagebiel R.W., Gail M.H., Elder D.E., Halpern A., A Holly E., Hartge P., A Tucker M. (2002). Average midrange ultraviolet radiation flux and time outdoors predict melanoma risk. Cancer Res..

[78] Solomon C.C., White E., Kristal A.R., Vaughan T. (2004). Melanoma and lifetime UV radiation. Cancer Causes Control.

[79] Wu S., Han J., Vleugels R., Puett R., Laden F., Hunter D.J., Qureshi A.A. (2014). Cumulative ultraviolet radiation flux in adulthood and risk of incident skin cancers in women. Br. J. Cancer.

[80] Dennis L.K., White E., McKnight B., Kristal A., Lee J.A., Odland P. (1996). Nevi and migration within the United States and Canada: A population-based cross-sectional study. Cancer Causes Control.

[81] Watson M., Geller A.C., Tucker M.A., Guy GPJr Weinstock M.A. (2016). Melanoma burden and recent trends among non-Hispanic whites aged 15–49 years, United States. Prev. Med..

[82] Garbe C., Leiter U. (2009). Melanoma epidemiology and trends. Clin. Dermatol..

[83] Weir H.K., Marrett L.D., Cokkinides V., Barnholtz-Sloan J., Patel P., Tai E., Jemal A., Li J., Kim J., Ekwueme D.U. (2011). Melanoma in adolescents and young adults (ages 15–39 years): United States, 1999–2006. J. Am. Acad. Dermatol..

[84] Guy GPJr Zhang Y., Ekwueme D.U., Rim S.H., Watson M. (2017). The potential impact of reducing indoor tanning on melanoma prevention and treatment costs in the United States: An economic analysis. J. Am. Acad. Dermatol..

[85] Colantonio S., Bracken M.B., Beecker J. (2014). The association of indoor tanning and melanoma in adults: Systematic review and meta-analysis. J. Am. Acad. Dermatol..

[86] Zhang M., Qureshi A.A., Geller A.C., Frazier L., Hunter D.J., Han J. (2012). Use of tanning beds and incidence of skin cancer. J. Clin. Oncol..

[87] Li W.Q., Cho E., Weinstock M.A., Mashfiq H., Qureshi A.A. (2016). Epidemiological assessments of skin outcomes in the nurses’ health studies. Am. J. Public Health.

[88] Li W.Q., Qureshi A.A., Ma J., Goldstein A.M., Giovannucci E.L., Stampfer M.J., Han J. (2013). Personal history of prostate cancer and increased risk of incident melanoma in the United States. J. Clin. Oncol..

[89] Schwartz M.R., Luo L., Berwick M. (2019). Sex Differences in Melanoma. Curr. Epidemiol. Rep..

[90] Giacomoni P.U., Mammone T., Teri M. (2009). Gender-linked differences in human skin. J. Dermatol. Sci..

[91] Oblong J.E. (2012). Comparison of the impact of environmental stress on male and female skin. Br. J. Dermatol..

[92] Damian D.L., Patterson C.R., Stapelberg M., Park J., Barnetson R.S., Halliday G.M. (2008). UV radiation-induced immunosuppression is greater in men and prevented by topical nicotinamide. J. Investig. Dermatol..

[93] Bedaiwi A., Wysong A., Rogan E.G., Clarey D., Arcari C.M. (2021). Arsenic Exposure and Melanoma among US Adults Aged 20 or Older, 2003–2016. Public Health Rep..

[94] National Research Council (1999). Arsenic in Drinking Water.

[95] Lothrop N., Wilkinson S.T., Verhougstraete M., Sugeng A., Loh M.M., Klimecki W., Beamer P.I. (2015). Home water treatment habits and effectiveness in a rural Arizona community. Water.

[96] Walker M., Seiler R.L., Meinert M. (2008). Effectiveness of household reverse-osmosis systems in a western us region with high arsenic in groundwater. Sci. Total Environ..

[97] Waypa J.J., Elimelech M., Hering J.G. (1997). Arsenic removal by RO and NF membranes. J. Am. Water Works Assoc..

[98] Vinceti M., Bassissi S., Malagoli C., Pellacani G., Alber D., Bergomi M., Seidenari S. (2005). Environmental exposure to trace elements and risk of cutaneous melanoma. J. Expo. Anal. Environ. Epidemiol..

